# Incidental Intraplacental Choriocarcinoma in the Setting of Intrauterine Fetal Demise

**DOI:** 10.1155/crog/3322909

**Published:** 2026-02-25

**Authors:** David K. Carlson, Gabrielle K. Smith, Robin D. LeGallo, Anne M. Mills, Kari L. Ring

**Affiliations:** ^1^ Department of Pathology, University of Virginia, Charlottesville, Virginia, USA, virginia.edu; ^2^ Department of Obstetrics and Gynecology, University of Virginia, Charlottesville, Virginia, USA, virginia.edu

**Keywords:** gestational trophoblastic neoplasia, intraplacental choriocarcinoma, IUFD, placental abnormalities

## Abstract

Gestational trophoblastic neoplasia (GTN) is a rare group of pregnancy‐related diseases involving the abnormal transformation of placental cells with metastatic potential. GTN includes choriocarcinomas, epithelioid trophoblastic tumors, placental site trophoblastic tumors, and invasive moles. The clinical presentation of GTN is often indolent and ultimately fatal if left untreated. Given the presentation of GTN and its low incidence, clinical care and proposed guidelines for managing GTN are limited, with a primary reliance on serum *β*‐hCG levels for treatment and surveillance. In this case report, we describe a case of intraplacental choriocarcinoma (IC) in the setting of intrauterine fetal demise (IUFD). We share the histopathologic diagnosis, the clinical management and surveillance for residual disease, and highlight the importance of placental examination for the purposes of building out clinical practice guidelines.

## 1. Introduction

Gestational trophoblastic neoplasia (GTN) is a group of pregnancy‐related diseases involving abnormal transformation and proliferation of placental cytotrophoblasts, syncytiotrophoblasts, and intermediate trophoblasts with metastatic potential. GTN includes choriocarcinomas, epithelioid trophoblastic tumors, placental site trophoblastic tumors, and invasive moles. GTN in the setting of a term pregnancy occurs in 1 in 150,000 cases [1, 2]. Malignant choriocarcinomas can be gestational or intraplacental. Although relatively rare, gestational choriocarcinoma (GC) is the most common GTN and can occur following a normal pregnancy in 1 in 50,000 cases [3]. Patients most often present with abnormal vaginal bleeding and amenorrhea. A widely described feature of GTN is elevated serum beta‐human chorionic gonadotropin (*β*‐hCG), and this marker is used for diagnosis, management, and surveillance [4, 5]. The true incidence of GTN is somewhat unknown given that placental examination is not routine in the setting of an unremarkable pregnancy course, and subsequently, GTN may go undiagnosed.

Intraplacental choriocarcinoma (IC) is one of the least frequently diagnosed forms of choriocarcinoma, with limited information available in the literature. IC is defined as choriocarcinoma originating in the placenta absent a primary uterine GC. An extensive systematic review of ICs published in 2016 by Sebire et. al identified only 62 reported cases of IC between 1986 and 2014 [6]. In their review, IC accounted for 0.03% of GTN (62/2,438). When clinical information was available, most women were diagnosed in the postpartum setting, had uneventful pregnancies, and were asymptomatic. Although the placentas were macroscopically unremarkable upon examination, some indications provoking placental examination included fetal/infant abnormalities, unexplained postpartum hemorrhage, and intrauterine fetal demise (IUFD). When comparing GC and IC, there is a clinical course deviation where GC is more likely to have symptoms of gestational bleeding, ultrasound findings, and macroscopic placental abnormalities. In the Sebire et al. review [6], the biologic behavior varied from local disease to metastasis. Although management with chemotherapy displayed a promising role in eliminating disease burden, especially in those with maternal or fetal metastatic disease, the indolent nature of IC compared with GC may result in increased morbidity and mortality. The authors were unable to ascertain baseline serum *β*‐hCG levels for the preexisting cases in literature, nor other diagnostic laboratory values that may detect abnormal trophoblastic activity.

In this current case report, we describe the clinical findings and ongoing management of a patient who experienced IUFD and later the diagnosis of IC in the setting of normal serum *β*‐hCG levels.

### 1.1. Case Summary

A 22‐year‐old G3P2001 female presented at 28w2d gestational age with a chief concern of decreased fetal movement. Her pregnancy was complicated by Factor V Leiden carrier status, bipolar disorder, asthma, as well as a prior tubal ectopic pregnancy and neonatal loss secondary to sudden infant death syndrome. Vital signs, blood count, and serum studies were within normal limits. During her pregnancy, she was taking a daily prenatal vitamin but no other prescribed medications. Prior ultrasound evaluations showed no fetal or placental anomalies. Type and screen revealed the patient is Rh B‐positive. Ultrasound evaluation at the time of emergency room presentation revealed the absence of fetal cardiac activity and confirmed the diagnosis of IUFD. No placental abnormalities were visualized at the time of IUFD diagnosis. Subsequently, the patient underwent induction of labor and delivered a nonviable female fetus. A fetal autopsy was declined by the patient.

**Figure 1:**
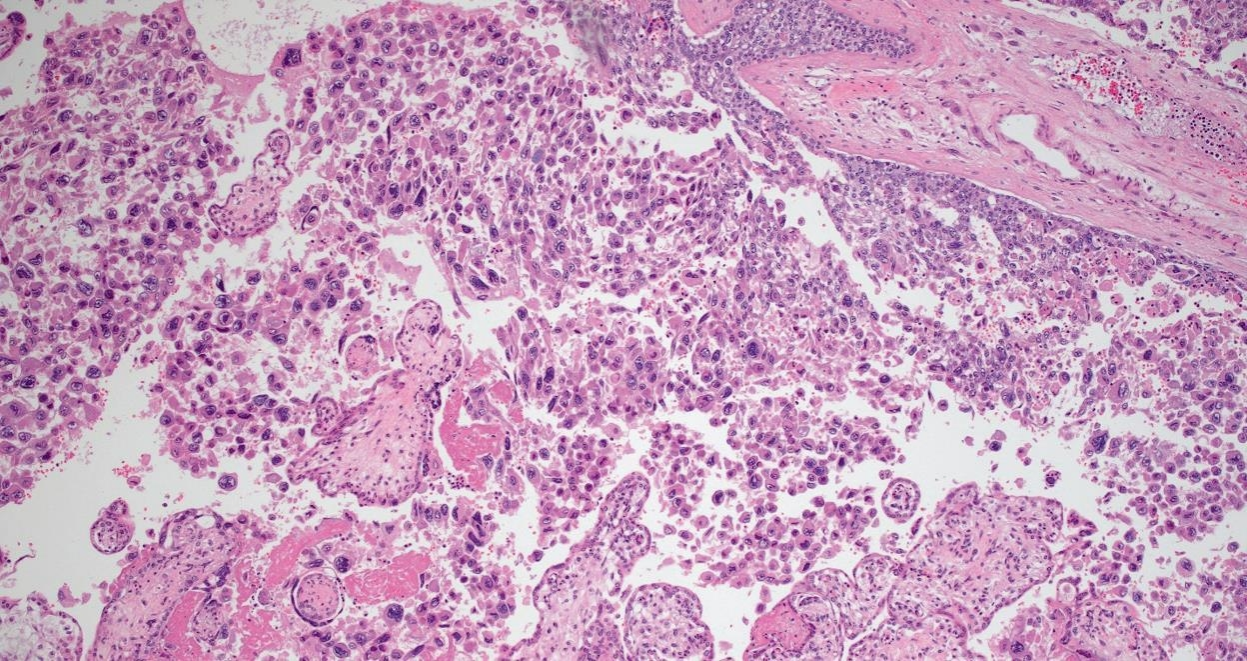
Intraplacental choriocarcinoma, 10× magnification: intraplacental choriocarcinoma adjacent to placental villi.

**Figure 2:**
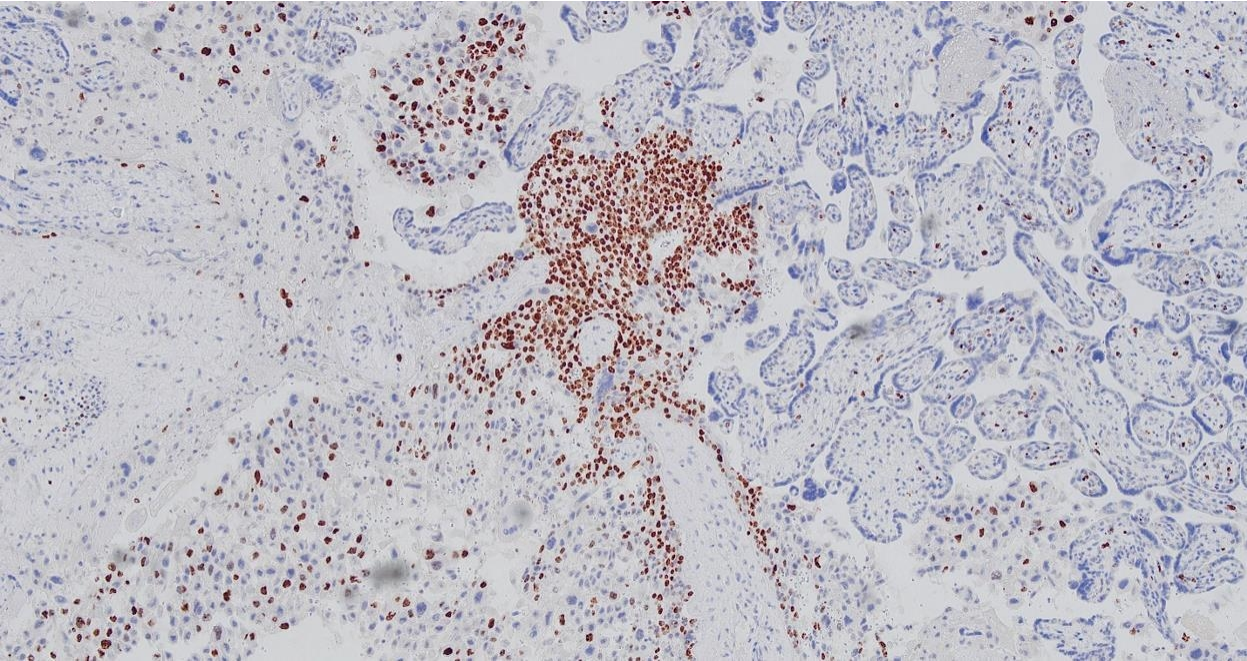
Intraplacental choriocarcinoma, 10× magnification, Ki‐67 stain: intraplacental choriocarcinoma demonstrating a high proliferation index (> 70%).

Per routine institution protocol following IUFD, all placentas are examined by anatomic pathology. Macroscopic examination of the placenta identified a centrally inserted hypocoiled three‐vessel umbilical cord (2 coils per 10 cm) measuring 10.7 cm in length. The symmetric placental disc measured 13.7 × 12.2 × 2.0 cm and weighed 260 g (an appropriate weight for gestational age). The placenta was serially sectioned into 2–3 cm thickness slices. The cut surfaces showed uniform, soft deep red placental parenchyma without any suspicious lesions or masses identified. Overall, the placenta appeared unremarkable. Microscopic examination of full‐thickness placenta parenchyma revealed a 12 mm × 8 mm focus of markedly atypical cytotrophoblasts, intermediate trophoblasts, and syncytiotrophoblasts with necrosis surrounding uninvolved villi (Figure 1). Immunostaining revealed strong expression of human placental lactogen (hPL) with focal admixed p63‐positive cells, whereas Ki67 showed a proliferative index of ~70% (Figure 2). Collectively, the morphologic and immunohistochemical findings supported the diagnosis of IC confirmed by pathologists with fellowship training in pediatric/perinatal pathology (R.D.L.) and gynecologic pathology (A.M.M.).

Following the finding of IC, a serum *β*‐hCG level performed 25 days after delivery was within normal limits at 2.4 U/L Imaging using computed tomography (CT) of the chest, abdomen, and pelvis, as well as transvaginal ultrasound, was negative for metastatic and residual disease, making the patient Stage I and low risk. A follow‐up appointment 1 month after delivery was notable for intermittent vaginal bleeding and depressed mood. A pelvic exam was performed at that time and demonstrated no evidence of vaginal or cervical metastases. After discussion with the patient and given the isolated lesion identified on microscopy, negative imaging, and normal *β*‐hCG, the patient elected to undergo surveillance with monthly clinic visits and monthly quantitative *β*‐hCG surveillance instead of chemotherapy. She chose to use medroxyprogesterone injections for contraceptive management during the surveillance period. After a 6‐month surveillance period, her monthly quantitative *β*‐hCG levels remained unelevated and within normal limits.

## 2. Discussion

This case report demonstrates a rare case of IC diagnosed on histopathologic placental evaluation in the setting of a workup following IUFD. Although IC is rare, it is a malignant GTN with metastatic potential. There are no definitive maternal, obstetric, or placental risk factors for the development of IC. Comorbidities, like Factor V Leiden carrier status, may influence placental pathology. For example, Factor V Leidein is associated with thrombophilia, placental infarction, and trophoblastic ischemia which may subsequently facilitate abnormal trophoblastic proliferation. To date, no such relationship has been established. Like other cases presented in literature, the current case received careful placental examination in the setting of IUFD. The relationship between IC and IUFD is not well understood; however, in the setting of maternal or fetal metastatic disease, the disease course is fatal if left untreated [6]. Current detection methods are primarily based on placental examination after delivery, usually in the setting of an abnormal pregnancy course or IUFD. In the presented case, IC was an incidental finding following an IUFD, with no grossly identifiable placental lesion and an unremarkable postpartum clinical course. In most cases of ICs, the maternal and fetal placental surfaces are normal appearing, with rare surface anomalies most frequently appearing similar to areas of fibrin deposition [7]. Additionally, the size of the intraplacental disease focus does not predict the degree of metastatic behavior [7]. Thus, IC largely remains a histopathologic diagnosis with potentially significant clinical consequences. It remains uncertain whether IC increases the risk of IUFD, or if IC is just more readily identified during reflexive placental examination following IUFD.

The clinical consequences of IC on pregnancy outcomes are difficult to assess given the rarity of the diagnosis. The disease can be largely asymptomatic, with about 60% of affected pregnancies resulting in live births [6]. When symptoms are present, vaginal bleeding is the most common [8]. IC is additionally associated with an increased risk of fetomaternal hemorrhage, fetal growth restriction, and stillbirth [8]. In cases of fetomaternal hemorrhage and IC, rates of perinatal mortality approach 37% [8]. In the presented case report, the relationship between incidentally found IC and IUFD is inconclusive; however, the previously available literature demonstrating fetomaternal hemorrhage and neonatal anemia in the setting of IC raises reasonable consideration that IC contributed to the clinical outcome of IUFD in this otherwise unremarkable pregnancy. To better elucidate the impact of IC on pregnancy outcomes, the true incidence of IC must be identified, which would require large scale, complete assessments of unselected placentas. This is logistically challenging given both the infrequency of the disease, as well as focality of IC on placental pathology. This suggests the providers should be thoughtful about requesting placental pathology in cases of unexpected pregnancy outcomes, even if routine placental exam in the delivery room is unremarkable, and, if IC is identified, complete a full staging workup regardless of the size of the disease focus.

Current standards for clinical care and proposed guidelines for managing IC are limited, mainly relying on serum *β*‐hCG and imaging to guide management. Specifically, there is a lack of data regarding management of IC in the setting of normal *β*‐hCG at time of diagnosis. Current FIGO and National Comprehensive Cancer Network (NCCN) guidelines advocate for administration of single‐agent chemotherapy for low‐risk GTN; however, the benefit of traditional chemotherapy in settings of nonmetastatic IC with normal serum *β*‐hCG levels is uncertain [9–11]. One retrospective cohort study out of Brazil investigated the utility of expectant management of patients with nonmetastatic IC with normal serum *β*‐hCG and found that only approximately half of these patients required chemotherapy, and that there were no differences in long term prognosis with delayed treatment [10]. In this case, our patient elected for expectant management and *β*‐hCG testing, rather than moving immediately to chemotherapy. Thus, this case adds important information regarding the safety of expectant management with B‐hCG monitoring in this population. IC generally has a low recurrence rate with appropriate treatment, and after completion of chemotherapy, patients frequently have good future pregnancy results [12].

Finally, given the rarity of cases, there is limited data to highlight recommendations for fetal monitoring during future pregnancies. It is known that treatment with EMA/CO (etoposide, methotrexate, actinomycin D, alternating with cyclophosphamide, and vincristine) substantially increases the risk of early menopause, which may impact future childbearing [13]. However, there is no known increase in incidence of congenital malformations in infants born to a patient who had previously received chemotherapy for GTN^13^. Patients should be counseled on an increased risk of miscarriage if conceiving within 6 months of completion of chemotherapy, although rates of stillbirth and preterm birth are thought to be unaffected [14]. However, there are no formal guidelines regarding a timeline or indication for initiation of antepartum monitoring, and further studies are needed to understand the impact prior IC may have on future pregnancy outcomes.

The case presented underscores the importance of placental examination. Routine histopathologic examination of the placenta following IUFD, even when ultrasound findings are unremarkable and the macroscopic appearance is normal is essential for detecting rare but clinically significant conditions like IC. At minimum, this aids in better understanding the clinical course of IC where limited clinical practice guidelines exist. More importantly, early recognition informs maternal evaluation and surveillance, potentially preventing a delayed diagnosis of metastatic disease.

## Author Contributions

**David K. Carlson:** writing – draft, review & edit. **Gabrielle K. Smith:** writing – draft, review & edit. **Robin D. LeGallo:** investigation, data curation, supervision. **Anne M. Mills:** writing – review & edit, supervision. **Kari L. Ring:** conceptualization, supervision.

## Funding

No funding was received for this manuscript.

## Consent

Written informed consent was obtained from the patient for publication of this case report and accompanying images. This case reports abides by ethical standards and ethical clearance is upheld.

## Conflicts of Interest

The authors declare no conflicts of interest.

## Data Availability

The data that support the findings of this study are available from the corresponding author upon reasonable request.
